# Supplementary material for: FERN – a Java framework for stochastic simulation and evaluation of reaction networks
Source: BMC Bioinformatics. 2008 Aug 29;9:356. doi: 10.1186/1471-2105-9-356 (PMC2553347; doi:10.1186/1471-2105-9-356)
Supplement: Additional file 1 — FERN distribution, Version 1.3. This archive contains the FERN source code and binaries as well as documentation and example models in FernML and SBML. [file 1471-2105-9-356-S1.zip › fern/doc/javadoc/fern/simulation/algorithm/package-frame.html]

fern.simulation.algorithm


fern.simulation.algorithm

Classes 
  
AbstractBaseTauLeaping
  
AbstractTauLeapingPropensityBoundSimulator
  
DependencyGraph
  
GibsonBruckSimulator
  
GillespieEnhanced
  
GillespieSimple
  
HybridMaximalTimeStep
  
IndexedPriorityQueue
  
TauLeapingAbsoluteBoundSimulator
  
TauLeapingRelativeBoundSimulator
  
TauLeapingSpeciesPopulationBoundSimulator
